# Bio-butanol production from glycerol with *Clostridium pasteurianum* CH4: the effects of butyrate addition and in situ butanol removal via membrane distillation

**DOI:** 10.1186/s13068-015-0352-6

**Published:** 2015-10-13

**Authors:** De-Shun Lin, Hong-Wei Yen, Wei-Chen Kao, Chieh-Lun Cheng, Wen-Ming Chen, Chieh-Chen Huang, Jo-Shu Chang

**Affiliations:** Department of Chemical Engineering, National Cheng Kung University, Tainan, Taiwan; Department of Chemical and Materials Engineering, Tunghai University, Taichung, Taiwan; Department of Seafood Science, National Kaohsiung Marine University, Kaohsiung, 811 Taiwan; Agricultural Biotechnology Center, National Chung Hsing University, Taichung, Taiwan; Research Center for Energy Technology and Strategy, National Cheng Kung University, Tainan, Taiwan

**Keywords:** Bio-butanol, *Clostridium pasteurianum*, Vacuum membrane distillation (VMD), Butyrate addition, Glycerol

## Abstract

**Background:**

*Clostridium pasteurianum* CH4 was used to produce butanol from glycerol. The performance of butanol fermentation was improved by adding butyrate as the precursor to trigger the metabolic pathway toward butanol production, and by combining this with in situ butanol removal via vacuum membrane distillation (VMD) to avoid the product inhibition arising from a high butanol concentration.

**Results:**

Adding 6 g L^−1^ butyrate as precursor led to an increase in the butanol yield from 0.24 to 0.34 mol butanol (mol glycerol)^−1^. Combining VMD and butyrate addition strategies could further enhance the maximum effective butanol concentration to 29.8 g L^−1^, while the yield was also improved to 0.39 mol butanol (mol glycerol)^−1^. The butanol concentration in the permeate of VMD was nearly five times higher than that in the feeding solution.

**Conclusions:**

The proposed butyrate addition and VMD in situ butanol removal strategies are very effective in enhancing both butanol titer and butanol yield. This would significantly enhance the economic feasibility of fermentative production of butanol. The VMD-based technology not only alleviates the inhibitory effect of butanol, but also markedly increases butanol concentration in the permeate after condensation, thereby making downstream processing easier and more cost-effective.

## Background

Glycerol is the principal byproduct of the biodiesel production process [1, 2], with production of ten gallons of biodiesel resulting in one gallon of glycerol byproducts. Since the biodiesel industry has been expanding rapidly in recent years, a large amount of glycerol has been produced, leading to a significant fall in its market price. The reutilization of glycerol by converting it into higher value products is thus of great interest. One of the options is using glycerol as a carbon source to produce butanol through fermentation processes.

Butanol is not only a key chemical in many industrial processes, but also an alternative fuel. It has several advantages over ethanol as a biofuel, in terms of energy content, volatility, hygroscopicity and the ease with which it mixes with gasoline in any proportion. Clostridia fermentation is known for its ability to produce butanol [3–5]. Acetone–butanol–ethanol (ABE) fermentation of sugars by *Clostridium acetobutylicum* is a well-known bio-butanol producing process and has been widely used in industry since the early 20th century. There are also several studies in the literature reporting reutilization of glycerol, a waste product of biodiesel manufacturing process, as the carbon source to produce butanol with the *Clostridium pasteurianum* strain [6–9].

Most butanol fermentation processes are inhibited by the accumulation of butanol in the fermentation broth, commonly known as “end-product inhibition” [10, 11]. The final butanol concentration in the fermentation broth is thus limited to a threshold (inhibitory) level. The inhibitory concentration of butanol is about 17 g L^−1^ for *C. pasteurianum* and 11–12 g L^−1^ for *C. acetobutylicum* [6, 12]. This is the primary factor impeding commercial acceptance of butanol production from renewable feedstock. Several separation techniques have thus been integrated with butanol fermentation processes for in situ solvent removal during batch and continuous butanol fermentation, and these include distillation, liquid–liquid extraction, adsorption by molecular sieves, and membrane separation [13–15]. To make butanol competitive with fossil fuels, the production costs must be reduced. Selective and continuous butanol removal from fermentation broth with a separation process can enhance the conversion rates, and consequently the economic feasibility of the butanol fermentation process [14]. The advantages of membrane separation methods, such as membrane distillation and pervaporation, include low energy demand, no removal of nutrients and substrates, no need for an entrainer, and a low possibility of contamination [16–18].

This study was undertaken to examine the efficiency of in situ butanol removal using vacuum membrane distillation (VMD) during the cultivation of *Clostridium pasteurianum* CH4, using glycerol as the carbon source. VMD is a promising technology for treating the aqueous solutions. The applications of VMD can be classified into three main fields: the single component transport process, the binary component transport process and the multi-components transport process, such as the desalination process and extraction of organic and dissolved gas from water. VMD has the potential of competing with other well-established separation technologies in terms of economic and safety considerations [19]. The relatively high energy demand required for the distillation is the major concern in the VMD operation. A possible solution to reduce the total energy consumption is to combine VMD operation with a heat recovery facility or to use renewable energy (such as solar energy) as part of energy supply.

In addition, the effect of adding butyrate at the beginning of fermentation (acting as a precursor in the butanol metabolism) on butanol production was also evaluated [20]. Finally, the combination of an in situ VMD module with the addition of butyrate was used to further enhance butanol production efficiency and obtain a higher glycerol utilization yield.

## Results and discussion

### Butanol separation performance with a vacuum membrane distillation process

**Fig. 1:**
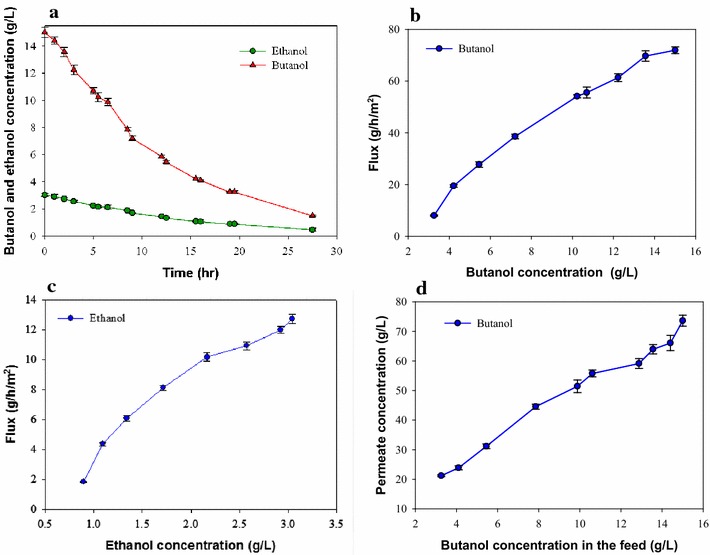
Removal of butanol and ethanol using the model BE solution. **a** The residual butanol and ethanol concentrations in the model BE solutions, **b** flux of butanol at various butanol concentrations, **c** flux of ethanol at various ethanol concentrations, and **d** performance of concentrated butanol concentration in the filtrate of vacuum membrane distillation (VMD). All the data presented here are mean values of duplicate experiments

To identify the selectivity of VMD on the separation of the main products of butanol fermentation (i.e., butanol and ethanol, denoted as BE), the rate of BE removal under the fermentation conditions was determined using prepared model solutions of B at a concentrations of 15.0 g L^−1^ and E at a concentration of 3.0 g L^−1^, respectively. The VMD system was used at 37 °C for the experiment. As shown in Fig. 1, with the VMD operating for 27 h the butanol concentration in the model solution decreased from 15 to 1.51 g L^−1^, while the ethanol concentration decreased from 3.0 to 0.47 (Fig. 1a). The effect of the feeding butanol and ethanol concentration on the rate of removal of butanol and ethanol was also investigated. Figure 1b shows that increasing the concentration of butanol in the feeding solution means increasing the mole fraction of butanol in the solution (or increasing the partial pressure of butanol), thereby leading to a higher butanol removal rate. The butanol flux with the VMD was 72 g h^−1^ m^−2^ at an initial butanol concentration of 15 g L^−1^. The effect of varying the ethanol concentration on the flux of ethanol removal was also investigated. As shown in Fig. 1c, the ethanol flux increased along with the concentration of ethanol. The ethanol flux with the VMD was 12.7 g h^−1^ m^−2^ when the ethanol feeding concentration was 3.0 g L^−1^. Using VMD, the butanol concentration in the permeate was up to five times higher than that in the feeding solution (Fig. 1d). The high concentration of butanol obtained in the permeate would facilitate the butanol recovery process. Overall, the results of the model solution clearly indicate that using the VMD system could efficiently remove butanol from the fermenter broth, thus greatly reducing the product inhibition that occurred in the ABE fermentation process.

### Effects of butyrate addition on batch butanol fermentation

**Fig. 2:**
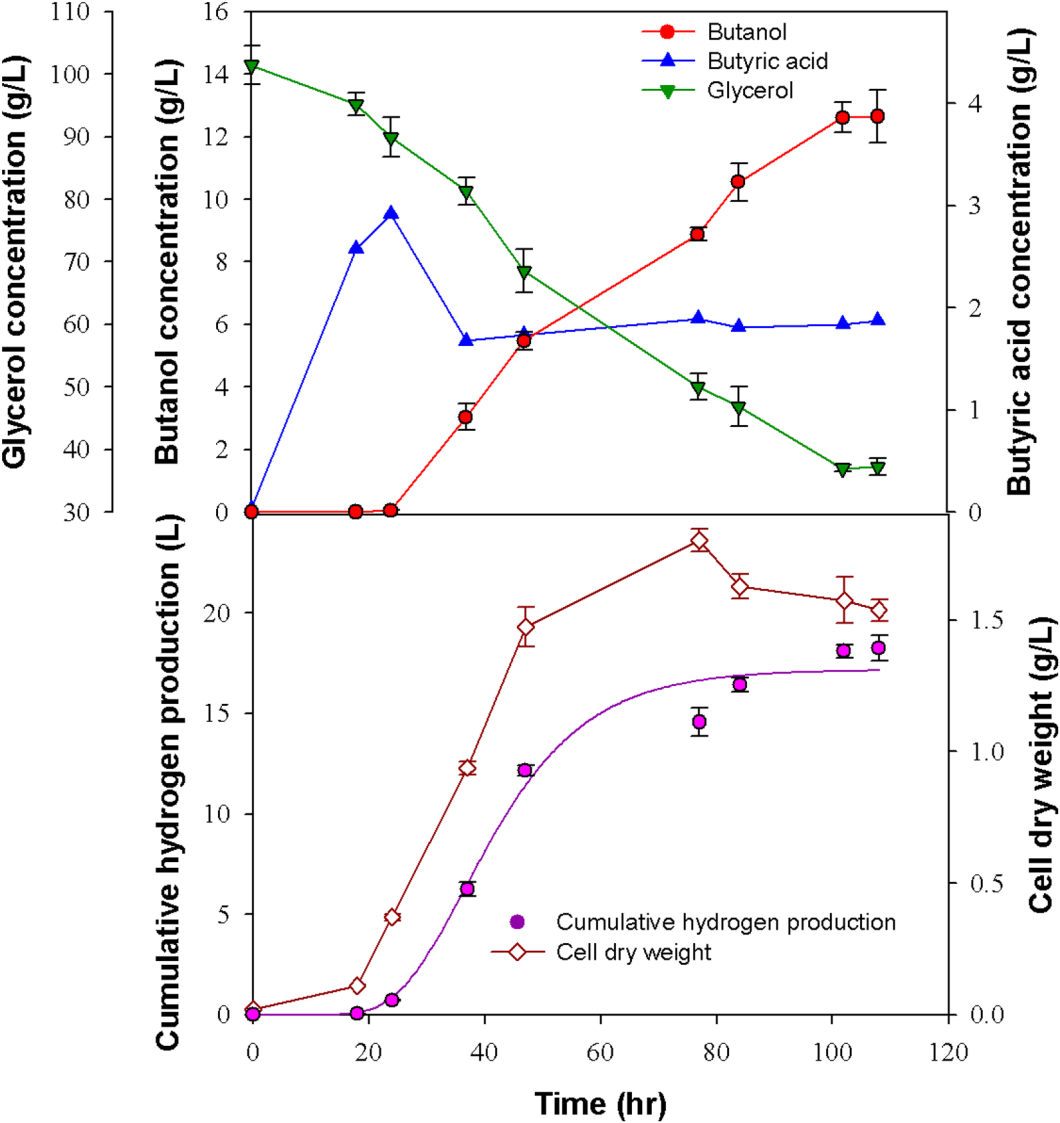
Batch production of butanol without the addition of butyrate and without integration with vacuum membrane distillation (VMD). All the data presented here are mean values of duplicate experiments

**Fig. 3:**
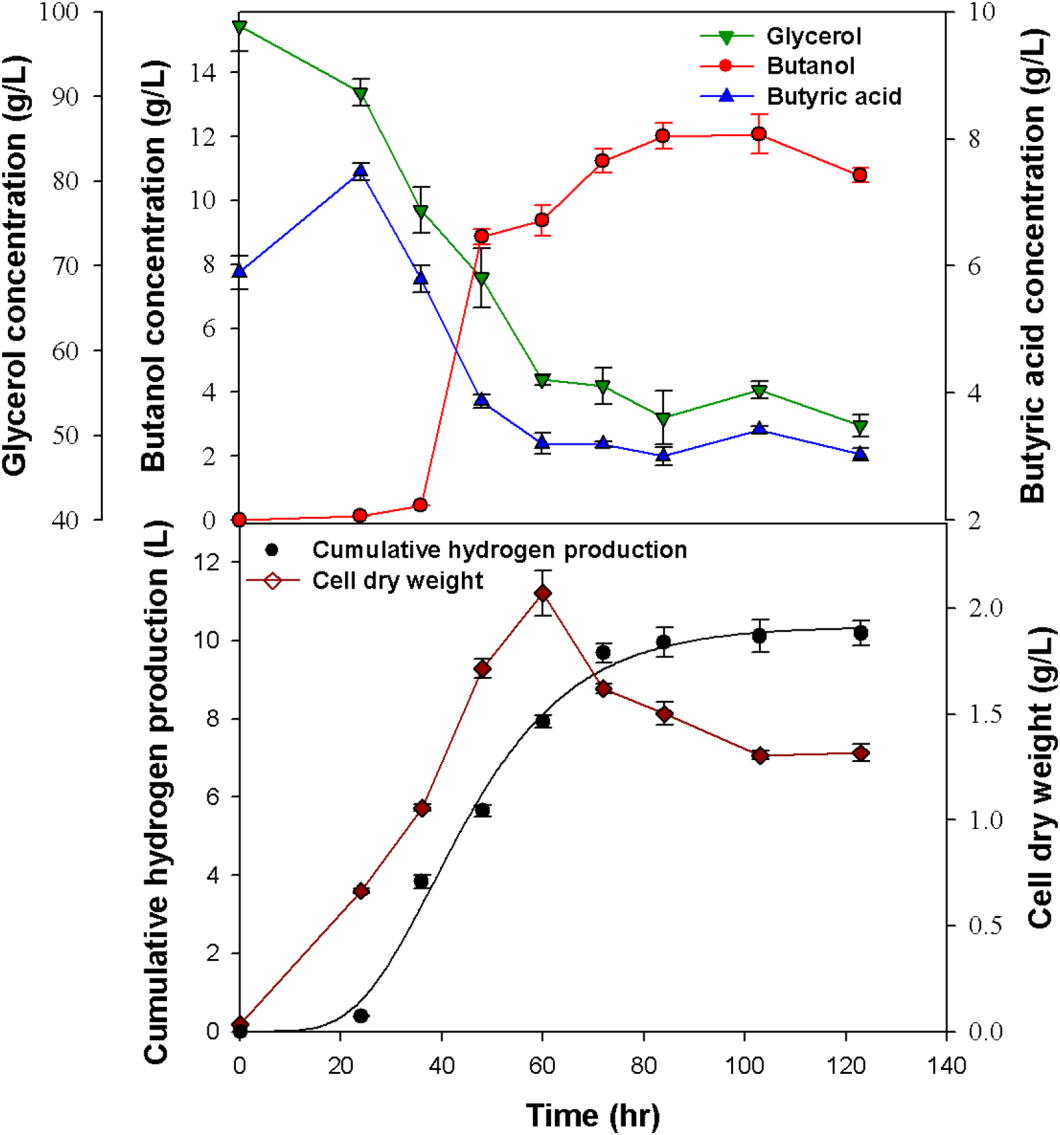
Batch production of butanol with the addition of butyrate, but without integration with vacuum membrane distillation (VMD). All the data presented here are mean values of duplicate experiments

Anaerobic fermentation has the potential to produce sustainable liquid and gaseous energy products from renewable feedstock. In this study, butanol and hydrogen were produced from glycerol with *C. pasteurianum* CH4 isolated from the effluent of a H_2_-producing bioreactor. In our preliminary work, response surface methodology (RSM) was adopted to investigate the optimum fermentation conditions, including the parameters such as culture pH, glycerol concentration and the added concentration of butyric acid. RSM is a useful tool to optimize a desired response when many factors and interactions are involved with the main advantage of reducing the number of experiments needed to evaluate multiple parameters and their interactions. Therefore, this tool is suitable for improving fermentation systems by optimizing the operating factors, which are in general very complex and interactive. The parameters used in this study (e.g., temperature, pH, glycerol concentration, medium composition, etc.) are based on this RSM optimization. However, this RSM analysis was not associated with VMD operation. The optimum conditions identified by the RSM analysis for the butanol production were as follows: temperature, 37 °C; pH, 5.5 (controlled); glycerol concentration, 100 g L^−1^; yeast extract concentration, 4 g L^−1^; FeSO_4_·7H_2_O concentration, 0.025 g L^−1^; and butyrate addition, 6 g L^−1^ (data not shown). Under these conditions *C. pasteurianum* CH4 cultivated in a 2 L fermenter without coupling with VMD and without addition of butyrate could produce a remarkable amount of H_2_ (i.e., 8.53 L H_2_ per liter of culture volume), with a yield of 0.45 mol H_2_/mol glycerol (Fig. 2). Meanwhile, the CH4 strain could produce butanol at a maximum concentration of 12.6 g L^−1^ and a yield of 0.24 mol butanol/mol glycerol. To further enhance butanol production in the 2 L fermenter, 6 g L^−1^ of butyrate was added into the medium at the beginning of the fermentation process as a precursor to stimulate butanol production. The maximum butanol concentration was 12.1 g L^−1^, with a yield of 0.34 mol butanol/mol glycerol, and the maximum hydrogen production was 5.18 L H_2_ L^−1^, with a yield of 0.41 mol H_2_/mol glycerol (Fig. 3). The results show that adding butyrate did not enhance the maximum butanol concentration, but instead the hydrogen production decreased (Fig. 3) and the butanol yield was higher. Since *C. pasteurianum* CH4 is capable of converting butyrate to butanol, when butyrate was added at the beginning of fermentation, the metabolic route of butyrate to butanol could be activated at the early stage. Butyric acid typically accumulates as an intermediate during the acidogenesis stage in the fermentation of *Clostridium* spp. The accumulated butyric acid can be utilized as the substrate for subsequent butanol production during the switch from acidogenesis to solventogenesis stage [20, 24]. The pathway from butyric acid to butanol is relatively short, not only from a metabolic point of view, but also with respect to the energy content. A successful uptake and further conversion of butyric could directly increase butanol yield. Hence, butyric acid is considered a desired and effective precursor to activate solventogenesis metabolism for butanol formation [20, 24]. The effect of butyrate on butanol production from sugars by *Clostridium* spp. has been widely reported, indicating that the addition of butyrate could effectively enhance butanol concentration and yield. [20, 24].

The early activation of the enzymes required for butanol formation triggered by adding butyrate into the fermentation broth seems to retard the metabolic pathway towards hydrogen production, and thus greatly improves the butanol production yield. It seems evident that, when butanol is synthesized from butyrate, reduced reagents are utilized for the reaction and as a consequence less H_2_ can be produced. Nevertheless, no further enhancement of maximum butanol production was observed with the addition of butyrate, due mainly to the feedback inhibition of high butanol accumulation. Therefore, to further increase butanol production, the VMD module was coupled with the fermenter to reduce the toxicity due to butanol accumulation in the fermentation broth. The results of VMD-integrated butanol fermentation are described in the following section.

### Effects of combining VMD with the addition of butyrate on butanol production

**Fig. 4:**
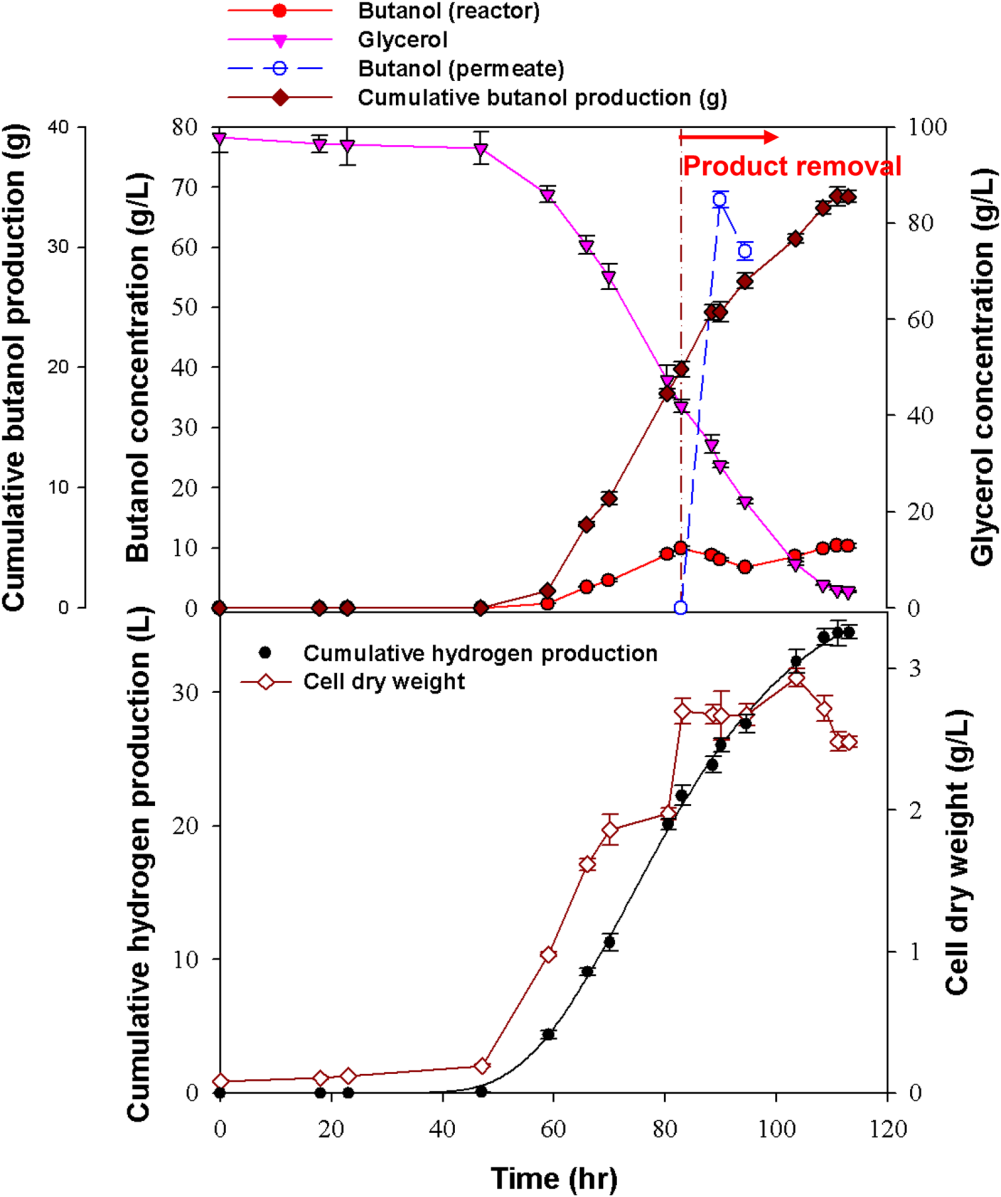
Production of butanol without adding butyrate but with VMD-integrated batch fermentation. All the data presented here are mean values of duplicate experiments

**Fig. 5:**
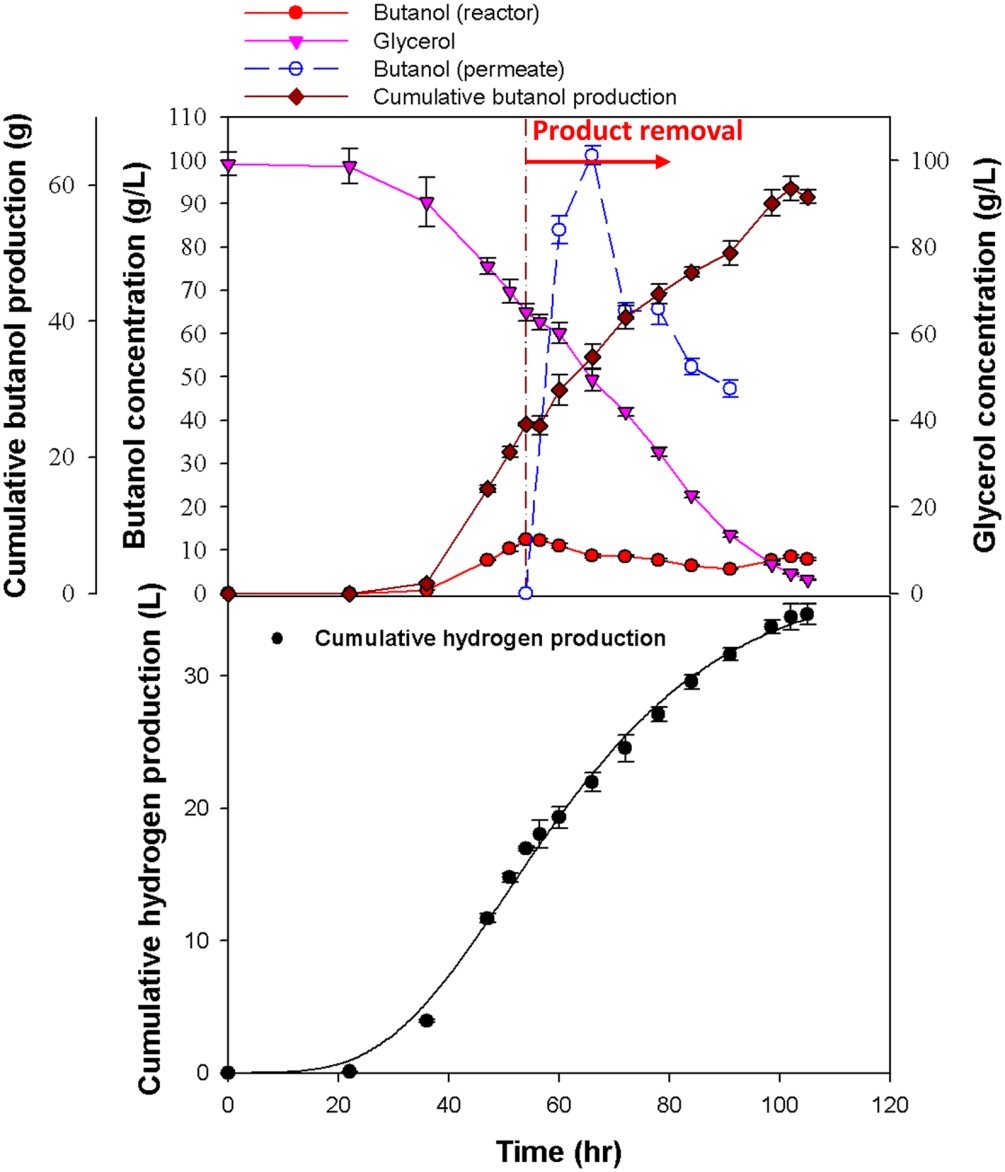
Production of butanol with the addition of butyrate as well as VMD-integrated batch fermentation. All the data presented here are mean values of duplicate experiments

The efficiency of in situ butanol removal via VMD combined with the addition of butyrate was examined in the batch butanol fermentation with *C. pasteurianum* CH4, using glycerol as the carbon source. Preliminary tests with synthetic aqueous solution of medium components and main products (i.e., glycerol, salts, ethanol, and butanol) show that water and butanol sufficiently moved across the membrane under the operational conditions. The results shown in Figs. 2 and 3 suggest that butanol inhibition occurred when the butanol concentration was higher than 12 g L^−1^. To alleviate the impact of end-product inhibition, the VMD system was connected to the fermenter and activated before butanol inhibition occurred (i.e., before the butanol concentration exceeded 10 g L^−1^). Figure 4 shows the results of in situ butanol removal via VMD during butanol fermentation without the addition of butyrate. The maximum final butanol concentration (17.1 g L^−1^) and butanol yield (0.23 mol butanol per mol glycerol) were achieved after fermentation for 111 h. As shown in Fig. 4, the butanol concentration in the permeate was 68 g L^−1^, while that in the fermentation broth was only 8 g L^−1^, indicating an 8.5-fold increase when the proposed VMD process was employed. In addition, the glycerol utilization efficiency was nearly complete (96.5 %) when the VMD was coupled with the fermenter, suggesting that the alleviation of butanol inhibition improved the substrate utilization efficiency. The VMD-coupled butanol fermentation was also combined with the butyrate addition strategy. Figure 5 shows that the maximum effective butanol concentration (29.8 g L^−1^) and butanol yield (0.39 mol butanol per mol glycerol) were obtained after fermentation for 102 h. The butanol concentration in the permeate was tenfold higher than that in the fermenter. In addition, the glycerol utilization also increased to 96.7 %.

Tables 1 and 2 summarize the kinetic information of biohydrogen and butanol production under different strategies, respectively. The estimated kinetic constants for biohydrogen production based on the modified Gompertz equation (Eq. 1) are presented in Table 1 [21–23]. This shows that the biohydrogen production was inhibited when butyrate was added, no matter with or without the use of VMD. Some researchers have reported that butyric acid is a critical precursor for butanol production, and addition of butyrate could enhance the butanol production and yield [20, 24]. Since butyrate is the precursor for butanol and could be a better substrate for conversion to butanol when compared with glycerol, poorer glycerol conversion was found when butyrate was added. The glycerol conversion decreased from 63.4 to 46.4 % when additional butyrate was added without the use of VMD (Table 2). However, when VMD was integrated with the fermentation process, both biohydrogen and butanol production were much higher than obtained with the non-VMD-integrated system, regardless of whether or not butyrate was added (Tables 1, 2). It is likely that the higher glycerol conversion of up to 96 % arising from integration of VMD may lead to the better performance of biohydrogen and butanol production. The biohydrogen and butanol production increased 2.5- and 3.6-fold, respectively, when compared with the non-VMD-integrated fermentation with butyrate addition (Table 2). The fermentation system with VMD was thus able to maintain a non-inhibitory level of butanol in the fermentation broth (Figs. 4, 5), and so the bacterium could function properly without the threat of toxicity arising from the inhibitory products (e.g., butanol), leading to the production of more biohydrogen and butanol.

**Table 1:** The estimated kinetic constants based on the modified Gompertz equation for hydrogen production with *C. pasteurianum* CH4 through batch fermentation using non-VMD-integrated and VMD-integrated processes with and without butyrate addition

	*λ* (h)	*H*_max_ (L L^−1^)	*R*_max_ (L L^−1^ h^−1^)	*R* ^2^	Hydrogen yield (mol H_2_/mol glycerol)
Non-VMD-integrated fermentation					
Without butyrate addition	25.0	8.53	0.30	0.985	0.45 ± 0.03
With butyrate addition	23.0	5.18	0.25	0.993	0.41 ± 0.04
VMD-integrated fermentation					
Without butyrate addition	55.0	19.55	0.39	0.999	0.78 ± 0.11
With butyrate addition	28.5	18.74	0.31	0.996	0.75 ± 0.06

*λ* lag time of H_2_ production (the time required for the onset of H_2_ production), *H*_*max*_ maximum H_2_ production estimated from the modified Gompertz equation, *R*_*max*_ maximum H_2_ production rate estimated from the modified Gompertz equation

**Table 2:** Butanol production with *C. pasteurianum* CH4 through batch fermentation using non-VMD-integrated and VMD-integrated processes with and without butyrate addition

Fermentation conditions		Butanol concentration (g L^−1^)	Glycerol conversion (%)	Average butanol production rate (g L^−1^ h^−1^)	Butanol yield (mol butanol/mol glycerol)
Non-VMD-integrated fermentation	Without butyrate addition	12.6 ± 0.8	63.4 ± 2.1	0.12 ± 0.02	0.24 ± 0.01
	With butyrate addition	12.1 ± 0.6	46.4 ± 0.5	0.14 ± 0.01	0.34 ± 0.04
VMD-integrated fermentation	Without butyrate addition	17.1 ± 0.3	96.5 ± 1.8	0.16 ± 0.02	0.23 ± 0.02
	With butyrate addition	29.8 ± 1.2	96.8 ± 0.9	0.29 ± 0.05	0.39 ± 0.01

The highest *H*_*max*_ (19.55 L L^−1^) and Y_H2_ (0.78 mol H_2_/mol glycerol) were obtained for VMD-integrated fermentation without butyrate addition, with these values being nearly double those obtained without the use of VMD (*H*_max_ = 8.53 L L^−1^, *Y*_H2_ = 0.45 mol H_2_ mol glycerol^−1^) (Table 1). The VMD-integrated system also had a higher biohydrogen production rate (*R*_max_ = 0.39 L L^−1^ h^−1^) than the non-integrated system (*R*_max_ = 0.30 L L^−1^ h^−1^). In addition to improving biohydrogen production, using the VMD-integrated fermentation strategy also markedly enhanced the butanol production when compared with the non-VMD-integrated system, regardless of whether butyrate was added or not (Table 2). These results suggest that removal of the inhibitory products (e.g. butanol) allowed nearly complete utilization of the substrate (i.e., glycerol), and thus improved butanol production. The VMD-integrated system with butyrate addition exhibited better butanol production performance, attaining a maximum effective butanol concentration of 29.8 g L^−1^, butanol production rate of 0.29 g L^−1^ h^−1^, glycerol utilization of 96.8 %, and butanol yield of 0.39 mol per mol glycerol. The results suggest that integration of VMD with the butanol fermentation system was effective in improving butanol production due primarily to the alleviation of butanol inhibition.

The comparison of Figs. 2, 3 with Figs. 4, 5 seems to show a significant increase of the bacterial growth lag phase duration when the VMD was integrated to the fermentation (Figs. 4, 5). The possible reason for a longer lag phase observed in the batch fermentation coupling with VMD could be due to the inadequate anaerobic condition when VMD was integrated. The initial empty space will increase while the fermentor was coupled with a VMD module. This may make it more difficult for the fermentor to maintain a strictly anaerobic condition, especially in the beginning of fermentation process, even though a longer N_2_ purging was employed. Therefore, improving the technique to create sufficient anaerobic condition would be necessary to reduce the lag phase during the VMD-coupled fermentation to further improve the butanol productivity.

Table 3 compares the performance of butanol fermentation using glycerol or a glucose-based carbon source. It shows that the efficiency of butanol fermentation obtained in this study with *C. pasteurianum* CH4 and glycerol, in terms of yield and substrate conversion, is higher than that obtained from most related studies. Comparing butanol fermentation from glycerol with that from glucose, the butanol yield from glycerol in this study (0.31 g butanol g glycerol^−1^) is comparable to that from glucose (0.27 g butanol g glucose^−1^) [25] (Table 3). However, the effective butanol concentration achieved in our system (29.8 g L^−1^) was lower than some cases shown in Table 3. Xue et al. [31] combined fed-batch fermentation and in situ product removal with gas stripping and achieved a high effective butanol concentration of 113.3 g L^−1^. However, it required a very high glucose concentration (475 g L^−1^) to achieve this high effective concentration of butanol. As a consequence, the butanol yield obtained from their system was only 0.24 g g glucose^−1^, which is lower than the yield obtained in this study (0.31 g g glycerol^−1^) using butyrate addition with VMD and an initial glycerol concentration of 100 g L^−1^. Moreover, although gas stripping is easy to operate and has been often used to remove volatile compounds (such as butanol) from liquid phase, it has the disadvantage of poor selectivity, high energy consumption, and generation of a large amount of water along with the removal of butanol. Table 3 also shows that using pervaporation for in situ removal of butanol is effective, also resulting in a high effective butanol concentration of 105.4 g L^−1^ [29]. However, to achieve this, an extremely high glucose concentration of 700 g L^−1^ was used. Also, the glucose utilization efficiency and butanol yield obtained from this system were both quite low, at 63.5 % and 0.24 g g^−1^ glucose, respectively [29] (Table 3). Pervaporation is a rapidly developing membrane technology and has the advantages of high selectivity and low energy demand. However, the characteristics of membrane would be critical to the successful operation of pervaporation and the cost of membrane is also relatively high. Minier et al. [32] also integrated fed-batch fermentation with ultrafiltration and distillation and achieved an effective butanol concentration of 41.5 g L^−1^, but again the butanol yield was quite low at only 0.16 g g glucose^−1^. Besides, using ultrafiltration and distillation for in situ butanol separation might be too costly. Therefore, based on the comparison mentioned above, the VMD-coupled butanol fermentation developed in this study seems to be an efficient and competitive butanol production method among the varieties of related systems reported. It was also found that the butanol tolerance of the fermentation bacterial strain using glycerol as a carbon source is slightly lower than that reported in an earlier work [6]. Therefore, enhancing butanol tolerance seems to be the next target to further improve the performance of butanol fermentation from glycerol.

**Table 3:** Comparison of the performance of butanol fermentation from glycerol and glucose with different *Clostridium* species

Strain	Carbon source	Butanol concentration (g L^−1^)	Yield (g butanol/g carbon source)	Carbon source utilization (%)	Fermentation strategy	References
*C. pasteurianum* DSM 525	Glycerol (170 g L^−1^)	3.33	0.12	16.2	Batch	[7]
*C. pasteurianum* DSM 525	Glycerol (114.6 g L^−1^)	14.3	0.2	55.5	Batch	[6]
*C. pasteurianum* ATCC 6013	Glycerol (25 g L^−1^)	5.8	0.24	96	Batch	[9]
*C. pasteurianum* DSM 525	Glycerol (25 g L^−1^)	8.7	0.36	96	Add lactate	[28]
*C. pasteurianum* CH4	Glycerol (100 g L^−1^)	12.6	0.20	63.4	Batch	This study
*C. pasteurianum* CH4	Glycerol (100 g L^−1^)	29.8	0.31	96.7	Butyrate addition and coupled with membrane distillation	This study
*C. acetobutylicum* ATCC 824	Glucose (700 g L^−1^)	105.4	0.24	63.5	Fed-batch and pervaporation	[29]
*C. beijerinckii* BA101 (mutant)	Glucose (59 g L^−1^)	11.9	0.27	75.3	Batch	[25]
*C. beijerinckii* BA101 (mutant)	Glucose (60 g L^−1^)	16.4	0.27	100	Gas stripping	[25]
*C. Saccharoperbutylacetonicum* N1–4	Glucose (50 g L^−1^)	14	0.31	89.4	Batch	[24]
*C. beijerinckii* P260	Straw hydrolysate (60 g L^−1^)	12.3	0.23	89	SHF	[30]
*C. acetobutylicum* JB200	Glucose (475 g L^−1^)	113.3	0.24	–	Fed-batch and gas stripping	[31]
*C. acetobutylicum* ATCC 824	Glucose (280 g L^−1^)	41.5	0.16	98	Fed-batch coupled with distillation and ultrafiltration	[32]

## Conclusions

This study demonstrated the feasibility of using glycerol as a carbon source for butanol production with an isolated strain *C. pasteurianum* CH4. The incorporation of the VMD system with the strategy of butyrate addition was very effective in enhancing butanol production, due to its capacity to maintain a non-inhibitory level of the toxic products in the fermentation broth, as well as achieve much higher glycerol conversion. The effective butanol concentration of the VMD-integrated system reached 29.8 g L^−1^ in batch fermentation, which is higher than most reported values. In addition, with the VMD system the butanol that is produced can be concentrated for easier downstream processing, and thus the proposed bio-butanol production process has considerable commercial potential.

## Methods

### Microorganism and culture medium

*Clostridium pasteurianum* CH_4_ isolated from effluent sludge of anaerobic H_2_-producing bioreactors [26] was adopted in this study as the butanol producer. The medium composition for the pre-culture was (g L^−1^): sucrose, 17.81; NaHCO_3_, 15; NH_4_Cl, 0.717; K_2_HPO_4_, 0.125; MgCl_2_.6H_2_O, 0.1; MnSO4.6H_2_O, 0.015; FeSO_4_·7H_2_O, 0.025; CuSO_4_·5H_2_O, 0.005; CoCl_2_·5H_2_O, 0.000125; CaCl_2_·2H_2_O, 0.1 [26]. The main culture medium contained the following components per liter of distilled water: glycerol, 40–100 g; NH_4_HCO_3_, 5.24 g; NaHCO_3_, 6.72 g; K_2_HPO_4_, 0.125 g; yeast extract, 4 g; casamino acid, 1 g; l-cysteine, 0.5 g; sodium thioglycolate, 0.5 g; MgCl_2_.6H_2_O, 100 mg; MnSO_4_·6H_2_O, 15 mg; CuSO_4_·5H_2_O, 5 mg; CoCl_2_·5H_2_O, 0.125 mg; FeSO_4_·7H_2_O, 25 mg. The culture temperature was 37 °C. Various amounts of butyrate were added to the medium separately in the experiments. In all experiments, the medium was sterilized at 121 °C for 20 min.

### Batch fermentation

A 2-L bioreactor was used throughout this study. In all experiments, the incubation temperature was controlled at 37 °C with a rotation rate of 100 rpm and an inoculum size of 1 % (v/v). The pH of the broth was controlled at 5.5. The addition of butyric acid 6 g L^−1^ was applied in the follow-up fermentation studies to enhance butanol production. The butanol and biohydrogen production were monitored during the time course of the experiment.

### Butanol production with in situ butanol removal via membrane distillation

**Fig. 6:**
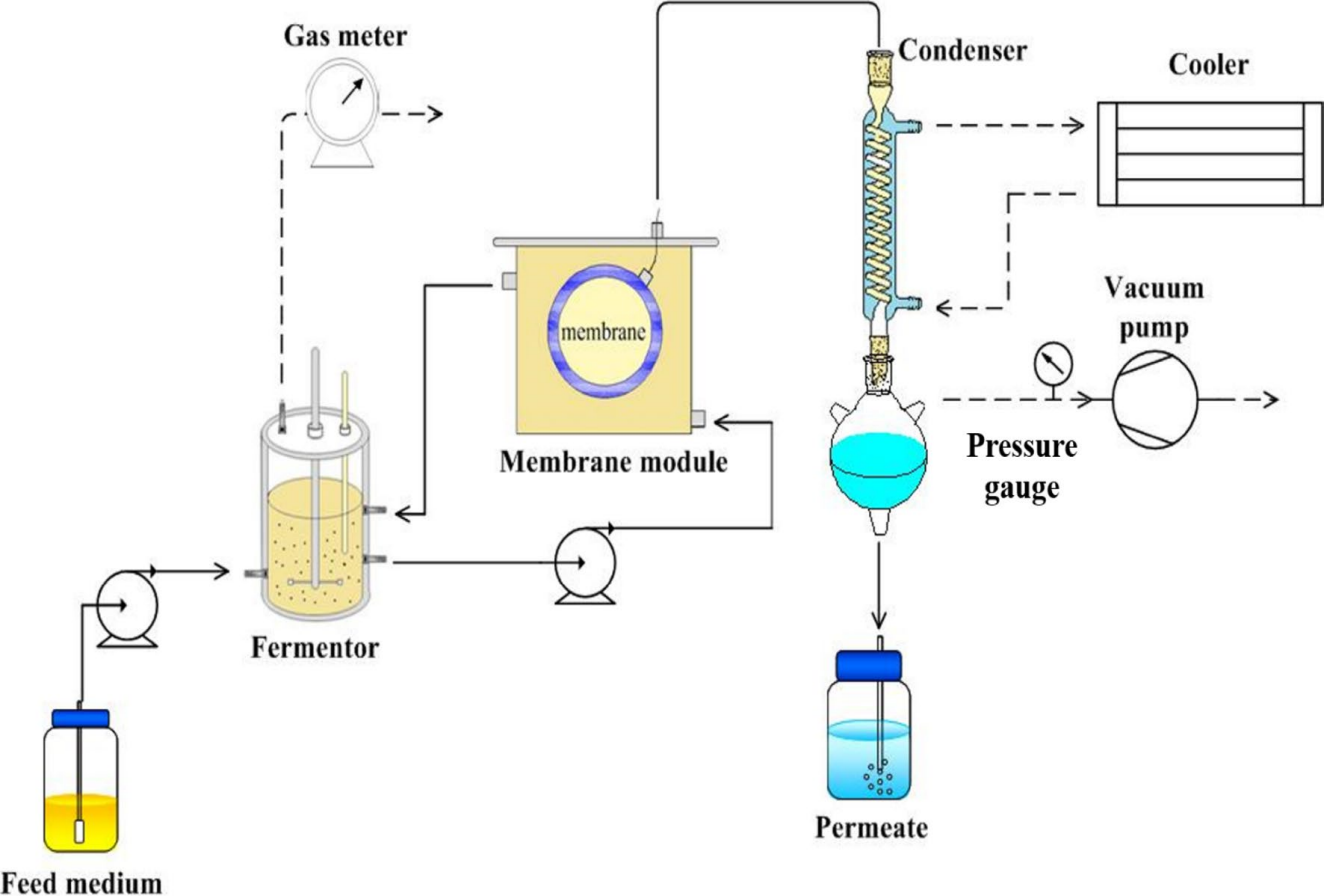
Scheme of butanol fermentation system coupled with VMD-based in situ butanol removal module

Butanol production using the strategy of butyrate addition was combined with VMD for the in situ separation of butanol from the fermentation broth to avoid product inhibition. The experimental setup is illustrated in Fig. 6. A fermenter with a working volume of 2 L was connected to a membrane distillation module. A capillary VMD module made with a polytetrafluoroethene (PTFE) membrane (King Membrane Energy Technology Inc, Tainan, Taiwan) was used in this study. This membrane has a pore size with a nominal diameter of 0.2 μm and a porosity of ca. 80 %. The effective membrane area amounts to 266 cm^2^. A vacuum was applied to create a driving force on the permeate side of the membrane module. The retentate was recycled back to the fermenter, while the permeate (distillate) was cooled by liquid nitrogen in a condenser. The effective butanol concentration achieved during the VMD-integrated butanol fermentation process was calculated as follows.

$${\text{Effective}}\,{\text{butanol}}\,{\text{concentration}}\, = \,\frac{{{\text{Sum}}\,{\text{of}}\,{\text{the}}\,{\text{amount}}\,{\text{of}}\,{\text{butanol}}\,{\text{in}}\,{\text{the}}\,{\text{broth}}\,{\text{and}}\,{\text{the}}\,{\text{permeate}}}}{{{\text{Initial}}\,{\text{volume}}\,{\text{of}}\,{\text{the}}\,{\text{fermentation}}\,{\text{medium}}}}$$

### Analytical methods

A high-performance liquid chromatography (HPLC) system equipped with a refraction index detector (RID-10A, Shimadzu, Japan) was used to detect soluble components (such as glycerol, acetate, butyrate, ethanol, and butanol) in the filtered (0.2 μm) supernatant of the culture broth. The column used in the HPLC analysis was an ICSep ICE-COREGEL 87H3 column (Transgenomic, USA). The mobile phase was 0.008 N H_2_SO_4_, with a flow rate of 0.4 ml min^−1^. The injection sample volume was 20 μl, and the column temperature was controlled at 55 °C. The gaseous products (H_2_ and CO_2_) were analyzed by gas chromatography (GC) using a thermal conductivity detector (China Chromatography, Taipei, Taiwan). The carrier gas was argon, and the column was packed with Prorapak Q [27]. The volume of H_2_ gas was measured by gas meter (TG1, Ritter Inc, Germany) at room temperature and 1 atm.

### Kinetic model: modified Gompertz equation

Since *C. pasteurianum* CH4 is also known as a biohydrogen producer, the control of the metabolic pathway toward to biohydrogen or butanol production is critical to enhancing the butanol concentration. To further clarify the interaction between biohydrogen and butanol production, a fitting model of a modified Gompertz equation was adopted for the measurement of biohydrogen production kinetic parameters. The Gompertz equation was proposed by Benjamin Gompertz in 1825, providing a good fit for the growth data of numerous tumors. Gompertz equation was successfully used for describing the growth of pure strains *Lactobacillus plantarum* from the early 1990s [27] and had been modified to estimate the cumulative hydrogen production in the anaerobic hydrogen production process [20, 24]. This equation was modified to describe the dynamic profile of biohydrogen based on the following two parameters: “potential maximum hydrogen production” and “maximum hydrogen production rate” [21]. Those can be easily obtained by the non-linear regression of the experimental data with the modified Gompertz equation (Eq. 1), shown below.

1 $$H = H_{\hbox{max} } \exp \left\{ { - \exp \left[ {\frac{{R_{\hbox{max} } \times e}}{{H_{\hbox{max} } }}\left( {\lambda - t} \right) + 1} \right]} \right\}$$

where *H*: cumulative H_2_ production (L L^−1^); *H*_max_: maximum H_2_ production (L L^−1^); *R*_max_: maximum H_2_ production rate (L h^−1^ L^−1^); *λ*: lag time (h); and *t*: incubation time (h).

## Abbreviations

VMD: vacuum membrane distillation
ABE: acetone–butanol–ethanol
BE: butanol and ethanol
PTFE: polytetrafluoroethene
HPLC: high-performance liquid chromatography
GC: gas chromatography

## References

[1] Ma FR, Hanna MA (1999). Biodiesel production: a review. Bioresour Technol.

[2] May YK, Tinia IMG (2011). A review of biodiesel production from *Jatropha curcas* L. oil. Renew Sust Energ Rev..

[3] Lee SY (2008). Fermentative butanol production by Clostridia. Biotechnol Bioeng.

[4] Bellido C (2015). Efficient acetone–butanol–ethanol production by *Clostridium beijerinckii* from sugar beet pulp. Bioresour Technol.

[5] Cheng HH (2015). Biological butanol production from microalgae-based biodiesel residues by *Clostridium acetobutylicum*. Bioresour Technol.

[6] Biebl H (2001). Fermentation of glycerol by *Clostridium pasteurianum*-batch and continuous culture studies. J Ind Microbiol Biotechnol.

[7] Dabrock B, Bahl H, Gottschalk G (1992). Parameters affecting solvent production by *Clostridium pasteurianum*. Appl Environ Microbiol.

[8] Kao WC (2013). Enhancing butanol production with *Clostridium pasteurianum* CH4 using sequential glucose-glycerol addition and simultaneous dual-substrate cultivation strategies. Bioresour Technol.

[9] Taconi KA, Venkataramanan KP, Johnson DT (2009). Growth and solvent production by *Clostridium pasteurianum* ATCC^®^ 6013TM utilizing biodiesel-derived crude glycerol as the sole carbon source. Environ Prog Sustain.

[10] Maddox IS, Qureshi N, Thomson KR (1995). Production of acetone–butanol–ethanol from concentrated substrate using *Clostridium acetobutylicum* in an integrated fermentation-product removal process. Process Biochem.

[11] Qureshi N, Maddox IS (2005). Reduction in butanol inhibition by perstraction: utilization of concentrated lactose/whey permeate by *Clostridium acetobutylicum* to enhance butanol fermentation economics. Food Bioprod Process.

[12] Branduardi P (2014). Microbial *n*-butanol production from Clostridia to non-Clostridial hosts. Eng Life Sci.

[13] Jeon YJ, Lee YY (1989). In situ product separation in butanol fermentation by membrane-assisted extraction. Enzyme Microbiol Technol.

[14] Vane LM (2008). Separation technologies for the recovery and dehydration of alcohols from fermentation broths. Biofuel Bioprod Biorefinery.

[15] Yen H, Chen Z-H, Yang I-K (2012). Use of the composite membrane of poly(ether-block-amide) and carbon nanotubes (CNTs) in a pervaporation system incorporated with fermentation for butanol production by *Clostridium acetobutylicum*. Bioresour Technol.

[16] Saketa Y (2011). Potable water recovery from As, U, and F contaminated ground waters by direct contact membrane distillation process. J Hazard Mater.

[17] Alklaibi AM, Lior N (2004). Membrane-distillation desalination: status and potential. Desalination.

[18] Zheng YN (2009). Problems with the microbial production of butanol. J Ind Microbiol Biotechnol.

[19] Chiam C-K, Sarbatly R (2013). Vacuum membrane distillation processes for aqueous solution treatment—a review. Chem Eng Process.

[20] Regestein L (2015). Impact of butyric acid on butanol formation by *Clostridium pasteurianum*. Bioresour Technol.

[21] Van GS, Sung SW, Lay JJ (2001). Biohydrogen production as a function of pH and substrate concentration. Environ Sci Technol.

[22] Lin CY, Lay CH (2004). Effects of carbonate and phosphate concentrations on hydrogen production using anaerobic sewage sludge microflora. Int J Hydrog Energy.

[23] Mu Y, Yu HQ, Wang G (2007). A kinetic approach to anaerobic hydrogen-producing process. Water Res.

[24] Tashiro Y (2004). High butanol production by *Clostridium saccharoperbutylacetonicum* N1-4 in fed-batch culture with pH-stat continuous butyric acid and glucose feeding method. J Boisci Bioeng.

[25] Ezeji TC, Qureshi N, Blaschek HP (2003). Production of acetone, butanol and ethanol by *Clostridium beijerinckii* BA101 and in situ recovery by gas stripping. World J Microbiol Biotechnol.

[26] Lee KS (2004). Anaerobic hydrogen production with an efficient carrier-induced granular sludge bed bioreactor. Biotechnol Bioeng.

[27] Cheng CL (2012). High yield biobutanol production by solvent-producing bacterial microflora. Bioresour Technol.

[28] Ahn J-H, Sang B-I, Um Y (2011). Butanol production from thin stillage using *Clostridium pasteurianum*. Bioresour Technol.

[29] Qureshi N (2001). Acetone butanol ethanol (ABE) recovery by pervaporation using silicalite-silicone composite membrane from fed-batch reactor of *Clostridium acetobutylicum*. J Membr Sci..

[30] Qureshi N (2008). Removal of fermentation inhibitors from alkaline peroxide pretreated and enzymatically hydrolyzed wheat straw: production of butanol from hydrolysate using *Clostridium beijerinckii* in batch reactors. Biomass Bioenerg.

[31] Xue C (2012). High-titer *n*-butanol production by *Clostridium acetobutylicum* JB200 in fed-batch fermentation with intermittent gas stripping. Biotechnol Bioeng.

[32] Minier M (1990). Extractive acetonobutylic fermentation by coupling ultrafiltration and distillation. Biotechnol Bioeng.

